# Ensuring vaccine safety: Case studies of falsified influenza vaccines detected in Brazil

**DOI:** 10.1016/j.jvacx.2023.100343

**Published:** 2023-07-01

**Authors:** Jaline Alves Cabral da Costa, Maria de Lourdes Aguiar-Oliveira, David Brown, Jorge Antonio Zepeda Bermudez, Eduardo Jorge Rabelo Netto, Ozéias de Lima Leitão, Antonio Eugenio Castro Cardoso de Almeida, Marilda Mendonça Siqueira

**Affiliations:** a National National Institute for Quality Control in Health (INCQS), Oswaldo Cruz Foundation, Av. Brazil, 4365, Manguinhos, Rio de Janeiro CEP 21040-360, Brazil; b Laboratory of Respiratory Virus and Measles, Oswaldo Cruz Institute, Oswaldo Cruz Foundation, Av. Brazil, 4365, Manguinhos, Rio de Janeiro CEP 21040-360, Brazil; c Sergio Arouca National School of Public Health (ENSP), Oswaldo Cruz Foundation, Av. Brazil, 4365, Manguinhos, Rio de Janeiro CEP 21040-360, Brazil

**Keywords:** Falsified vaccine, Influenza vaccine, Brazil, Quality control, Public health

## Abstract

Falsifications related to health technologies—including vaccines—are a growing threat to patient safety and health systems on a global scale and can cause serious harm to the population (especially vulnerable groups). In Brazil, the manufacturing and spread of counterfeit medicines are prevented through joint actions between different government agencies. In this study, we analyzed three cases of influenza vaccines suspected of counterfeiting. The samples were seized by officials and received by the National Institute for Quality Control in Health (INCQS), the national quality control reference laboratory of the Ministry of Health of Brazil, in 2010, 2017, and 2020. We report the results of our analytical investigations and emphasize the importance of strengthening the partnerships between various national agencies. The seized samples were visually inspected, and their information was compared with that of genuine vaccines (as recorded in the INCQS database). The specific analytical tests were based on quality control tests for biological products. Our results confirmed that all seized samples were falsified. We emphasize the importance of fostering international and intra-national collaborations between various national agencies (such as drug regulatory authorities, official laboratories, customs departments, police forces, and civil society). As demonstrated here, such collaborative actions are essential for combating the release of falsified medical products, safeguarding public health, and strengthening health systems.

## 1 Introduction

Vaccines have been established as effective tools for reducing the burden of infectious diseases, and this fact has been highlighted in the key role played by vaccines in confronting the Covid-19 pandemic. However, like other health technologies, vaccines are also subject to fraud, and this is a serious problem for public health and the economy worldwide [1], [2], [3], [4], [5]. Over the years, this problem has been discussed in several public forums, especially those related to medicine [6], [7], [8], [9], [10]. Supply–demand imbalances and the increased vulnerability of global supply chains has created opportunities for organized criminal groups to introduce falsified medicines into the marketplace. Vaccine hoarding—which has been severely criticized during the Covid-19 pandemic, especially when practiced by wealthy economies—may impose an additional burden on developing economies because of inequality, leading to counterfeiting [11], [12]. Furthermore, the growth of e-commerce and illegal online practices poses a particular hazard, especially when combined with the general public's lack of awareness about the associated risks [13], [14], [15]. Saliou et al. described this growing phenomenon of fake vaccine trafficking and pointed out that any vaccine can be falsified [16].

The administration of these counterfeit products to patient can have serious consequences, including treatment failure and disease aggravation. Moreover, exposure to unknown and unsafe substances can cause serious damage to one’s health and even lead to death [2], [17]. Specifically, the use of counterfeit vaccines can lead to ineffective population immunization, a decline in vaccine coverage, and the undermining of patient confidence in immunization programs and health systems [16], [18], [19]. Thus, it is essential to fight against the falsification of medicines. In this sense, controlling these activities is a global challenge that requires the implementation of dynamic and interrelated strategies aimed at effective prevention, detection, and response [20], [21].

The guidelines of the World Health Organization (WHO) suggest that prevention consists of avoiding the sale and consumption of counterfeit products, ensuring the quality of the products, and guaranteeing the integrity of the supply chain. Prevention can be improved by implementing education programs to raise consumer awareness about the risks of using counterfeit drugs. To detect counterfeits, it is necessary to provide inspection agencies with access to analytical laboratories with adequate technical capacity and enhancements. Finally, the WHO guidelines indicate that a quick and proportional response to any counterfeiting of medicines—including vaccines—is crucial for protecting public health and preventing recurrence [17], [22].

Unlike drugs with a chemically defined composition and structure, vaccines are more complex and difficult to manufacture. Therefore, the quality control of vaccines is performed through specific tests on each batch. These tests are conducted according to the standards defined in official compendia, pharmacopeia monographs, and the WHO Technical Reports Series [23], [24]. Thus, only batches with assured quality and safety are distributed for use within the health system [25]. Suspected counterfeit vaccines are inspected visually and their information is verified, and specific laboratory tests are conducted to identify counterfeit vaccines under WHO recommendations. It is essential to identify and quantify the vaccine's active ingredient [26], and this determination is usually performed through a biological assay [24].

All these analyses unequivocally require technical and scientific knowledge and expertise, and the effectiveness of this system guarantees the quality of the vaccine for use by the population, as well as ensuring the success of the investigation in cases of possible fraud [23], [24].

Among the vaccines available for vaccination, the influenza vaccine is widely used worldwide. Annually, influenza viruses of genera A (Alphainfluenzavirus) and B (Betainfluenzavirus) cause seasonal epidemics of varying severity can result in substantial disease burden [27], [28]. These viruses are enveloped and contain two major glycoprotein antigens, hemagglutinin (HA) and neuraminidase (NA) inserted. Influenza A viruses are further classified into subtypes according to their glycoprotein HA (18 subtypes) and glycoprotein NA (11 subtypes), while influenza B viruses are further classified into two lineages: B/Victoria/2/87 and B/Yamagata/16/88 [27], [29]. Most inactivated trivalente or quadrivalent vaccines used in Brazil and worldwide are manufactured in embryonated eggs or cell cultures, submitted to the split process, inactivation by formalin, without the use of adjuvant. These vaccines contain influenza A virus strains, H1N1 and H3N2 subtypes, and influenza B virus (one or two strains), as listed annually by the World Health Organization. Standardization of influenza vaccines is based on hemagglutinin content (80% of the viral surface structure after the shedding process), with most vaccines containing 15 μg/0.5 mL of each hemagglutinin antigen [27].

In this study, we highlight the problem of vaccine counterfeiting by identifying and examining cases of falsified influenza vaccines detected in Brazil in 2010, 2017, and 2020. Our findings suggest that the National Institute for Quality Control in Health (INCQS), the National Control Laboratory (NCL) of the Brazilian Ministry of Health, could increase its involvement in the investigation of other falsified vaccines or medicines to strengthen the fight against counterfeit products.

In this context, we detail the process for evaluating the falsified influenza vaccines detected in Brazil and highlight the contribution of the INCQS to the analytical investigation of vaccines suspected of falsification. Our findings reinforce the importance of strengthening the partnerships between various national government agencies in the struggle against vaccine counterfeiting, as this can reduce overall expenses and speed up the investigation process. This problem is not restricted to Brazil and is common in countries where regulatory agencies impose technical and scientific restrictions. As such, there is an urgent need for large-scale mobilization against the growing global threat of vaccine falsification.

## 2 Methods

In this study, we present three cases of influenza vaccines suspected of falsification. The reports were received by the INCQS in 2010, 2017, and 2020, and the samples were seized by official agencies for analytical investigation. We received 3 samples for case 1, 41 for case 2, and 22 for case 3 (Table 1). The scientific team at INCQS determined the investigative strategy for the seized vaccine samples based on WHO recommendations and pharmacopeial monographs [23], [30]. The investigation was conducted in three stages: i) visual inspection of the seized material, which can reveal signs of vaccine adulteration; ii) verification of vaccine information against the INCQS database and supporting reference materials; and iii) specific analytical tests using samples from each case. Visual inspection and information verification were performed for all samples, and analytical tests were performed only for samples with adequate volume.

**Table 1:** Description of the influenza vaccine samples in each case under suspicion of falsification.

**Case/ Samples (Year)**	**Description of received samples and number of units**	**Code of the sample(s) used for analytical tests**
Case 1 3 samples (2010)	Pre-filled single-dose syringe with the label, secondary packaging, and package insert:	
	− 03 units containing ∼ 0.5 mL of an opaque liquid	A
Case 2 41 samples (2017)	Multi-dose vial with a rubber stopper with perforation marks:	
	− 01 unlabeled vial with ∼ 2 mL of a colorless liquid	B
	− 03 unlabeled vials with a colorless residue	–
	Multi-dose vial with an intact metal seal:	
	− 01 unlabeled vial with ∼ 5 mL of a clear colorless substance	C
	− 02 vials with damaged labels and ∼ 5 mL of a clear colorless substance	D, E
	Common syringe with a nominal capacity of 3 mL:	
	− 02 unidentified units with ∼ 0.5 mL/syringe of a clear colorless solution	F, G
	− 31 unidentified units with a colorless residue	–
	Pre-filled single-dose syringe with a label in Spanish:	
	− 01 unit with a colorless residue	–
Case 3 22 samples (2020)	Red-labeled vial (batch X):	
	− 01 vial with a colorless residue	–
	− 03 vials with ∼ 5 mL of a clear colorless	H
	Yellow-labeled vial (batch Y):	
	− 11 vials containing ∼ 5 mL of a clear colorless solution	I
	− 02 vials containing ∼ 5 mL of a clear colorless liquid with visible black particulate matter	–
	Vial with a red label (batch Z):	
	− 05 used vials containing ∼ 1 mL of a clear colorless liquid	J

### 2.1 Visual inspection

During the visual inspection, all samples were evaluated in terms of appearance, clarity, opacity, and presence of contamination. The type of pharmaceutical presentation (pre-filled single-dose syringe or multi-dose vial) was also recorded and verified against the manufacturer's original sanitary surveillance records. For samples contained in vials, the presence or absence of the metallic seal and the appearance of the rubber cap were evaluated. All labels, the secondary packaging, and leaflets were carefully evaluated. We also checked the information provided in the labels and evaluated the layout and print color of the printed details [26], [31]. The final dataset included the batch number, manufacturing data, and expiry date of the seized samples, the manufacturer's name and address, and information regarding the registration number. These details were compared with those of genuine vaccines and with reports provided by the manufacturer.

### 2.2 Database—the laboratory sample management system

In the second stage of the investigation, we consulted the INCQS database in the laboratory sample management system (Harpya) to verify all available information about the seized samples. This database is a national information system for managing the samples of healthcare products [32]. This system records all information regarding the products submitted for sanitary analysis, starting from the receipt of the sample at the INCQS to the issuance of the Certificates of Analysis. In addition, this database contains all results related to the quality control analyses of each lot of medicinal product used throughout the country [33]. It also records information related to vaccines and their manufacturers with a high degree of reliability.

In this step, the results obtained from the visual analysis were compared with the database records, and the authenticity of the seized samples was verified. The official documents of genuine vaccines and reports provided by the manufacturers were also consulted. Additionally, we contacted the Federal Police, sanitary surveillance agencies, and the manufacturers of the original vaccines to facilitate the exchange of information on the seized materials.

### 2.3 Analytical tests

The laboratory tests used in the analytical investigation were defined based on the quality control tests for biological products, which are available from the INCQS and are recommended by the Brazilian pharmacopoeia [23]. Before conducting these tests, we took into account the available volume of the seized samples and previous requests from official agencies.

#### 2.3.1 Evaluation of potency and identity

Priority was given to determining the pharmaceutically active ingredient through the single radial immunodiffusion (SRD) assay, which is a gold-standard potency assay for influenza vaccines [34]. This test measures the concentration of hemagglutinin antigen (HA) based on the *in vitro* reaction of specific antigens and antibodies, such that each dose should contain at least 15 μg HA/0,5mL per strain [35], [36].

For these tests, we used specific reference standard antigens and antisera defined for each strain (H1N1, H3N2, and B) and for each season (as provided by the National Institute for Biological Standards and Control). These reference standards are produced and calibrated by the WHO Essential Regulatory Laboratories and are distributed both to manufacturers and to the National Control Laboratories, which perform independent control tests of vaccine batches [36]. Samples of genuine monovalent influenza vaccines—previously approved by the INCQS—were used as positive controls in these assays.

To complement the investigation process, additional analytical tests were performed, including quantification of the total protein, residual formaldehyde, and aluminum content. All assays were performed in accordance with the Brazilian pharmacopoeia [23].

#### 2.3.2 Protein measurement

The total protein content was estimated by ultraviolet absorption (specification: ≤300 µg/0.5 mL). Proteins contain tyrosine and tryptophan side chains, which are strong absorbers of light in the 275- to 280-nm (ultraviolet) region. Consequently, after suitable dilution to produce on-scale absorbance readings, total protein can be estimated from UV absorbance spectra using quartz or fused silica cuvettes [23]. A 250 to 300 nm run was performed using a blank sample (ultrapure water), positive control samples, and samples under investigation, with a sufficient volume to perform the test without sample dilution. The absorbance was measured using a Varian Cary 50 Spectrophotometer. Genuine trivalent influenza vaccine (previously approved by the INCQS) was used as the positive control.

#### 2.3.3 Formaldehyde measurement

Formaldehyde is a substance commonly used as a viral inactivator in influenza vaccine formulations. Residual formaldehyde was measured by means of titration and spectrophotometric analysis of the solution after reaction with 2.5% trichloroacetic acid solution and Hantzsch reagent. By reacting traces of formaldehyde with an approximately neutral solution of acetylacetone and ammonia, a yellow color gradually develops, characteristic of the synthesis of diacetyldihydrolutidine [23]. The residual formaldehyde in the blank sample, standard formaldehyde solution (Standard/1 and Standard/2) and the samples was determined by measuring the absorbance using a Varian Cary 50 Spectrophotometer (412 nm, specification: ≤30 µg/ individual human dose).

#### 2.3.4 Aluminum measurement

The aluminum content (specification: ≤1.25 mg/individual human dose) was determined only in seized samples with an opaque appearance (case 1) [23]. All spectrophotometric measurements were made using a PerkinElmer Optima 8300 ICP-OES Spectrometer and WinLab32 version 6.2.0.0079. Samples of genuine pneumococcal vaccine and genuine diphtheria, tetanus toxoid, and pertussis vaccine (which contain aluminum hydroxide as an adjuvant; previously approved by the INCQS) were used as positive controls.

## 3 Results

### 3.1 Results of visual inspection and verification against the INCQS database

The visual analysis of the seized samples revealed several irregularities in terms of the printed batch number, registration number, manufacturing and expiry dates, and the layout and print color of details printed on label and outer packaging. Attempts to verify these details against vaccine registration requirements and the INCQS database revealed strong evidence of falsification.

#### 3.1.1 Analysis of the visual appearance of seized samples

**Fig. 1:**
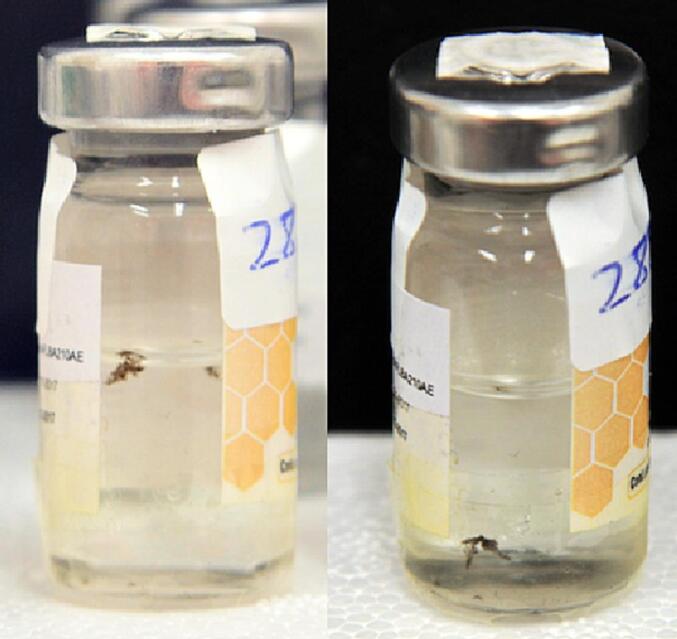
Vials with visible black particles matter, indicating the presence of contaminants (case 3).

In cases 2 and 3, 22 samples consisted of a colorless liquid substance. In addition, two samples in case 3 also had visible black particles matter, indicating the presence of contaminants (Fig. 1).

**Fig. 2:**
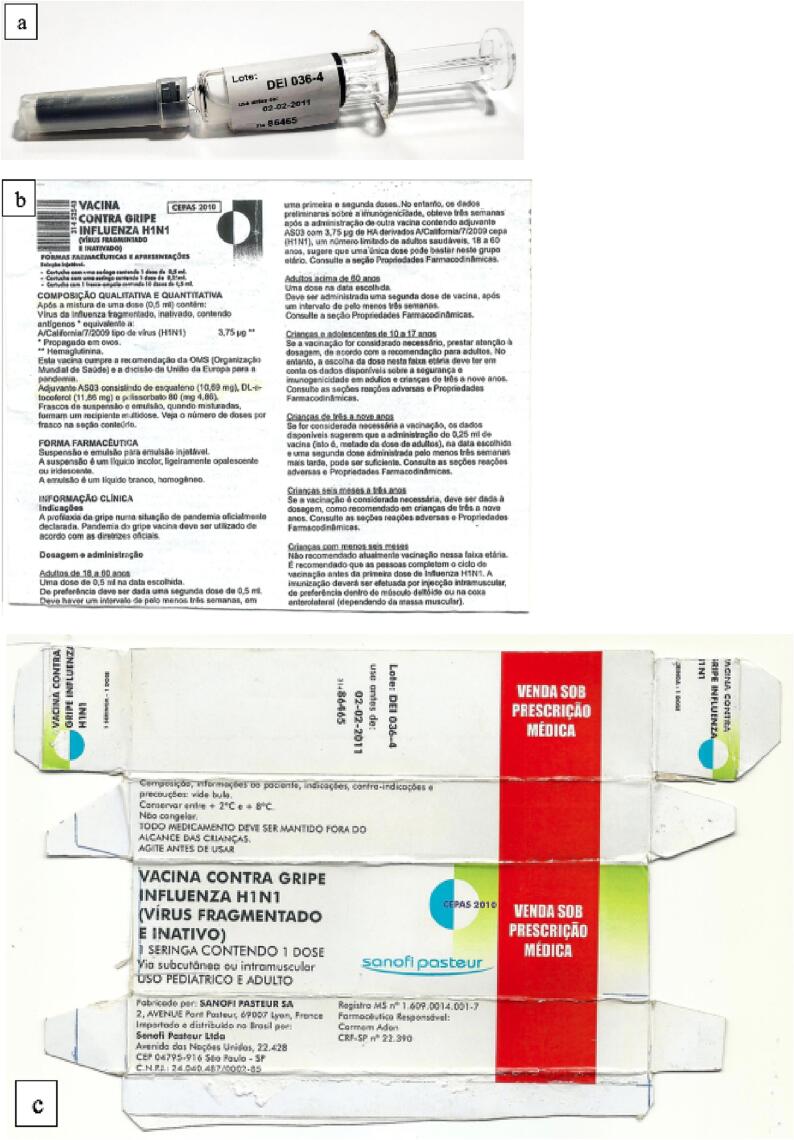
Pharmaceutical presentation of samples seized in case 1. (a) Pre-filled Single-dose syringe. (b) Package inserts. (c) Secondary packaging.

In case 1, three seized samples had an opaque appearance (Fig. 2a) and were markedly different from the original vaccine (which has a clear appearance). This opaque appearance indicated the presence of aluminum hydroxide, a widely used adjuvant that potentiates the immune response of the antigen. However, the manufacturing process of the genuine influenza vaccine does not involve the use of any adjuvants.

#### 3.1.2 Pharmaceutical presentation and volume of seized samples

The seized samples had different pharmaceutical presentations (pre-filled single-dose syringes or multi-dose vials). Overall, 5 samples contained a reduced volume of the seized substance, and 41 samples consisted of a colorless liquid. Case 1 only included samples in a pre-filled single-dose syringes with a package insert and external packaging (Fig. 2a-c).

**Fig. 3:**
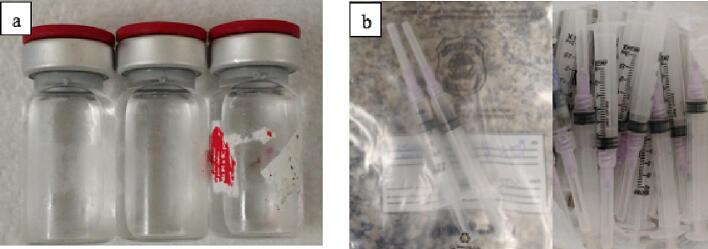
Pharmaceutical presentation of samples seized in case 2. (a) Multidose vials with missing or damaged labels. (b)* Common syringes with a nominal capacity of 3 mL containing approximately 0.5 mL of liquid and used syringes with residues of substances. Source: *Reproduction of image by the Brazilian Federal Police.

In case 2, samples in multidose vials and one sample in a pre-filled single-dose syringe were seized from a clandestine clinic. The vials were very damaged or had missing labels (Fig. 3a), and some vials had a damaged metal seal and scratched or perforated rubber caps. In addition, we also found common syringes with a nominal capacity of 3 mL containing a clear and colorless solution and syringes with residues of substances, indicating strong evidence of clandestine immunization activity (Fig. 3b).

**Fig. 4:**
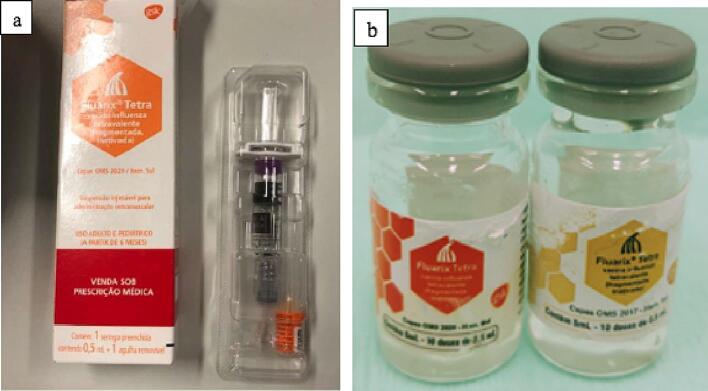
Pharmaceutical presentation of samples seized in case 3. (a)* The original vaccine, manufactured and marketed only in pre-filled single-dose syringes and never in multidose vials. (b) The counterfeit vaccine in multidose vials with labels in different colors (red and yellow) for the same product, including inconsistencies in layout and information (batch number, registration number, manufacturing and expiry dates). Source: *Reproduction of image by the National Health Regulatory Agency of Brazil. (For interpretation of the references to color in this figure legend, the reader is referred to the web version of this article.)

In case 3, we found that the pharmaceutical presentation of the multidose vials did not correspond to the original vaccine records. The genuine vaccine was only produced and marketed in pre-filled single-dose syringes containing 0.5 mL of the vaccine, and never in a multidose vial. In addition, the vials had labels in different colors for the same product (Fig. 4b).

#### 3.1.3 Analysis of secondary packaging, package inserts, and labels

Samples seized in case 1 were the only ones with secondary packaging and leaflets. All these details were rigorously inspected. We observed several signs of counterfeiting in the secondary packaging, including changes in the color pattern of the packaging; absence of a security seal (that guarantees the inviolability of the product); and absence of the reactive ink mechanism (which identifies the logo of the vaccine company). In addition, these packages had a handmade appearance, featuring lines made with a ballpoint pen and manually cut boxes (Fig. 2c). Similar indicators of counterfeiting were also observed in the package inserts. We identified spelling mistakes and found poor-quality printing that resembled copies made on common printers (Fig. 2b). We also found that the counterfeit leaflet contained a partial transcription of data from the leaflet of another influenza vaccine manufacturer. A report from Sanofi Pasteur (a vaccine manufacturer) confirmed these inconsistencies. This report contained a checklist comparing the data of the original vaccines with those associated with the counterfeit vaccines. Other indications of falsification were also identified on the labels; for example, the batch number, manufacturing/expiry dates, and recommended vaccine strain composition of the seized samples did not match the corresponding details in the original vaccine records (Fig. 2a, Fig. 4b).

Among the samples seized in case 2, we identified a pre-filled single-dose syringe with a label in Spanish. We evaluated the characteristics of the label and syringe, and this product showed no signs of counterfeiting. However, cross-checking the information on the label (such as the batch number and manufacturer details) revealed that this product was intended for marketing only in a specific country in South America, and not in Brazil.

In case 3, two indications of fraudulence were detected in the same seizure. In addition to the inconsistencies in pharmaceutical presentation, the labels on the vials had been printed in different colors (red and yellow) for the same product. We also found inconsistencies in the print layout and other details (such as the batch number, registration number, and manufacturing and expiry dates) (Fig. 4a-b).

### 3.2 Results of analytical tests

The results of the laboratory tests (Table 2) corroborated the suspicions at initial diagnosis. All samples submitted for potency (evaluated by the presence or absence of a halo) and identity, both evaluated by vaccine antigen-antisera reaction defined for each strain (H1N1, H3N2, and B) tests through the single radial immunodiffusion (SRD) assays, showed negative results, indicating the absence of a pharmacologically active substance.

**Table 2:** Analytical tests on samples of influenza vaccines under suspicion of falsification.

**Sample**	Potency test*	Identity test*	Aluminum content*	Total protein content*	Residual formaldehyde*
A	Absence of halo	Negative	0.43 mg/0.5 mL	ND	ND
B to G	Absence of halo	Negative	ND	ND	ND
H, I	Absence of halo	Negative	ND	Negative	Negative
J	ND	ND	ND	Negative	ND

ND = not done. *Specifications: potency test: ≥15 µg/0.5 mL; identity test: positive/negative; aluminum content: ≤1.25 mg/0.5 mL; total protein content: ≤300 µg/0.5 mL; residual formaldehyde: ≤30 µg/0.5 mL.

In addition, the assays for residual formaldehyde and total protein content (performed only on samples from case 3) showed no absorbance peak over threshold, indicating the absence of these compounds in the samples. Samples with an opaque appearance suggested the presence of adjuvants such as aluminum hydroxide (case 1). For these samples, the investigation was complemented with an analysis of aluminum dosage. This assay detected aluminum at a concentration of 0.43 mg/0.5 mL (Table 2), indicating marked differences with the original vaccine (which has a clear appearance and does not contain any adjuvants). The positive controls in all tests presented results compatible with the specifications.

## Discussion:

In this study, we found evidence of vaccine falsification, handmade packaging, package inserts, and labels. We also detected the presence of an aluminum-based adjuvant, which is not part of the composition of the genuine influenza vaccine. In addition, we found evidence indicating the commercialization of a product that is not authorized by the country's regulatory agency, suggesting possible smuggling activity. According to the police, one Brazilian and three Lebanese people were involved in setting up a clandestine clinic to administer H1N1 influenza vaccines acquired in a country bordering Brazil. During the seizure, law enforcement officials seized not just the falsified vaccines, but also weapons, ammunition, and silencers; the group was charged with drug trafficking [37].

In our study, the laboratory analysis of the seized samples mainly focused on the identification and quantification of the pharmaceutically active ingredient in each sample [26], [38]. As noted, the negative results obtained for the potency and identity assays confirmed the absence of pharmacologically active substances. Furthermore, the residual formaldehyde and total protein content assays also indicated the absence of these compounds in the samples. Thus, all these results indicate a strong potential for falsification. Another relevant issue was the presence of microbial contamination in a sample of seized vaccines, which could have serious consequences for health both individually and collectively. Furthermore, the risks of toxicity and failure to prevent the disease targeted by the vaccine can lead to the uncontrolled spread of an epidemic and even death to the multiplication of victims [16].

The material evidence from these analytical tests is essential for assessing whether a crime has been committed with implications for public health.

The results obtained in this study made it possible to launch a criminal investigation in which offenders can receive prison sentences of up to 15 years (in addition to fines and administrative sanctions) [39], [40]. In addition, the National Health Regulatory Agency (ANVISA) issued alerts for the seizure and destruction of all irregular vaccine lots throughout the country [41], [42].

The results of this study clearly indicate the presence of counterfeit vaccines in the Brazilian market. Although a high level of control is required to stop these illicit activities, the dissemination and permanence of fraudulent vaccines is also facilitated by certain features of the current system. These include the high economic benefit for criminals at the expense of weak penal sanctions (that is, high profit with low risk); absent or ineffective national regulatory authorities; limited access to safe medical products with satisfactory quality; and high prices and globalization of the pharmaceutical market [1], [2], [26]. The increasing complexity of supply chains also facilitates the introduction of counterfeit products [43].

In this context, counterfeit vaccines can also have serious health-related consequences, including providing a false sense of security against a dangerous virus and potentially loss of confidence in science, authorities, and reliable medicines. Furthermore, the proliferation of counterfeiting also has business-related effects such as disincentive innovation, discourage investments in research and development activities, and undermine intellectual property system [44].

According to the WHO, falsified medical products are those whose identity, composition, or source has been altered in a deliberate and fraudulent manner (2). These ineffective products—that often contain no active ingredients—are made available in markets and replace the necessary vaccines. This type of falsification causes serious socio-economic harm and has negative impacts on global public health [17], [45]. To encourage countries to report incidents of vaccine falsification, the WHO provides a structured reporting system and technical support in cases of emergency. It also facilitates international information exchange regarding issues and alerts related to the spread of substandard and falsified medical products [2].

In Brazil, preventing and combating the counterfeiting of medicines involves joint actions between the ANVISA, the Brazilian Federal Police (BFP), the INCQS, and other government agencies [46]. Given the relevance of the problem of vaccine counterfeiting, the Brazilian vaccine regulatory authority has been improving its sanitary inspection protocols and is working intensively to inhibit such illegal activities. The ANVISA participates in the Steering Committee of the Member State mechanism on substandard and falsified medical products under the purview of the WHO [47]. In accordance with WHO recommendations, the ANVISA has also developed a system for issuing alerts to facilitate the seizure and destruction of irregular products throughout the national territory [41], [42].

The BFP is responsible for investigating crimes related to the counterfeiting, corruption, and adulteration of medicines. It is also involved in effectively repressing smuggling. In addition, Federal Criminal Experts receive technical training on a regular basis and perform forensic analyses to confirm possible falsifications or irregularities [40]. The INCQS also participates in this process by responding to any requests for assistance in the analytical investigation of medicines that are suspected of falsification and seized by official agencies.

The INCQS is the national reference laboratory for the quality control of products of the Brazilian Ministry of Health. It also works closely with the national health surveillance system and aims to guarantee the quality, efficacy, and safety of medical products intended for the general population [48], [49]. It is the only official laboratory that certifies the release of all vaccines lots used in the country—whether by the National Immunization Program of the Ministry of Health or by the private sector (imported products)—and exported (in the case of WHO-prequalified vaccines) [25], [50].

Although various detection technologies are available in BFP laboratories, vaccines present additional technological challenges due to their complex nature. Vaccines contain one or more antigenic substances, and their analysis requires the use of specific immunobiological assays, reagents, and reference materials [24]. In cases of counterfeit biological products, the INCQS laboratory is capable of providing a qualified response and can perform adequate analysis [25]. The technical and scientific capacity of the INCQS allows it to meet the specific requirements for the quality control of biological products and consequently perform the diagnosis of counterfeit influenza vaccines.

Thus, the INCQS provides important tools that can contribute to the investigation of—and fight against—the falsification of vaccines and other health technologies.

It is important to emphasize the value of cooperation between the various public bodies involved in this study. The close exchange of information resulted in positive actions and rapid responses in the fight against vaccine counterfeiting. In this context, the dynamics reported in this study highlight the need to strengthen public policies related to the verification and control of medical products, including vaccines. Combating the falsification of these products requires the sharing of responsibilities between global organizations, national health authorities, the pharmaceutical industry, research institutions, and civil society. Evidently, the faster the exchange of information between these agencies, the better and more agile the evaluation system will be.

We also emphasize the importance of investing in equipment and the continuous training and qualification of technical teams, which enables the implementation of specific analytical systems. Regulatory systems can be strengthened by implementing effective policies, such as issuing alerts, enforcing legislation, and quickly identifying vulnerabilities in global supply chains [21], [22]. Additional measures that can help ensure the quality and safety of medical products include intensifying risk-based inspection and surveillance activities, implementing tracking technologies, connecting notification systems, and enforcing customs laws [47]. Given the considerable increase in falsification cases around the world, such efforts have become even more relevant. Much of this is due to the advent of internet-based facilities and the popular demand for medical products [3], [46].

The public disclosure of safety alerts and reports is essential for raising public awareness of the risks associated with counterfeit medicines. Therefore, authorities should strive to maintain a system of cooperation between regulatory and customs authorities, police forces, judicial authorities, and other stakeholders at the national level. This would encompass the most effective means of combating the trafficking of falsified medical products and addressing their impacts on society [17], [21].

Studies like this one can help in the investigation of other falsified vaccines or medical products, providing the population with access to safe, effective, and quality medical technologies. Studies such as this one are also crucial for ensuring compliance with the Federal Constitution of Brazil, which states that health is the right of all persons and a duty of the State. Additional findings can also help meet the challenges associated with achieving global health equity, human rights, and sustainable development goals.

## Funding

This work was supported by Oswaldo Cruz Foundation, Ministry of Health (MoH).

## Declaration of Competing Interest

The authors declare that they have no known competing financial interests or personal relationships that could have appeared to influence the work reported in this paper.

## Data Availability

Data will be made available on request.
